# Gingivo-Periodontal Alterations in Pediatric Leukemia: A Comparative Analysis of IL-1β, IL-6, IL-17α and TGF-β1 Levels in Gingival Crevicular Fluid and Plasma

**DOI:** 10.3390/dj13100450

**Published:** 2025-09-30

**Authors:** Alina Adumitroaie, Larisa Ghemiș, Vasilica Toma, Ancuta Goriuc, Oana Tanculescu, Melissa Entuc, Simina Vacariu, Liliana Georgeta Foia

**Affiliations:** 1 Department of Pediatric Dentistry, Grigore T. Popa University of Medicine and Pharmacy Iasi, Universitatii Street 16, 700115 Iasi, Romania; alina.adumitroaie@umfiasi.ro (A.A.);; 2 Department of Biochemistry, Grigore T. Popa University of Medicine and Pharmacy Iasi, Universitatii Street 16, 700115 Iasi, Romania; larisa.danaila@umfiasi.ro (L.G.); ancuta.goriuc@umfiasi.ro (A.G.); georgeta.foia@umfiasi.ro (L.G.F.); 3 Department of Fixed Prosthesis, Grigore T. Popa University of Medicine and Pharmacy Iasi, Universitatii Street 16, 700115 Iasi, Romania; oana.tanculescu@umfiasi.ro; 4 Department of Medical Sciences II, Grigore T. Popa University of Medicine and Pharmacy Iasi, Universitatii Street 16, 700115 Iasi, Romania; 5 Division of Medical Analyses Laboratory, Clinical Emergency Hospital St. Spiridon, 700111 Iasi, Romania

**Keywords:** children, leukemia, gingivo-periodontal alterations, inflammation, cytokines

## Abstract

**Background/Objectives**: Our study aimed to evaluate the oral health status associated with levels of certain inflammatory biomarkers in the gingival crevicular fluid (GCF) and plasma of children and adolescents with leukemia, in comparison to healthy subjects, in order to assess the correlation between pediatric leukemia and gingivo-periodontal alterations. **Methods**: The study was conducted on 97 subjects, divided into two groups: the study group, *n* = 47 leukemia subjects, and the control group, *n* = 50 healthy subjects. The collected GCF and plasma samples were analyzed for interleukins IL-1β, IL-6 and IL-17α and transforming growth factor TGF-β1 values using ELISA (enzyme-linked immunosorbent assay) techniques. **Results**: IL-6, IL-17α, and TGF-β1 recorded higher values for leukemia subjects, both in the GCF and plasma, compared to healthy subjects. Our results also pointed out higher gingival fluid IL-1β values in children with leukemia compared to the control group. **Conclusions**: Elevated expressions of IL-1β in the GCF and IL-6, IL-17α, and TGF-β1, both systemic and local, in the GCF of children with leukemia were associated with oral hygiene status and gingival inflammation, respectively. All inflammatory biomarkers generally tended to rise in close correlation with oral hygiene worsening and gingival inflammation extension; however, IL-1β in the GCF and plasma, plasma IL-6, and gingival fluid IL-17α pointed out a stronger correlation with gingival inflammation status.

## 1. Introduction

The literature highlights an increased incidence of mucosal pathologies and a predominance of gingivo-periodontal involvement among the oral manifestations observed in children affected by leukemia [1]. These can be caused either by the disease itself, which triggers a cascade of systemic and local changes, or by the antineoplastic treatment, which suppresses the immune system and predisposes the individual to xerostomia and mucosal tissue alterations, alongside direct effects of certain medications upon the gingival tissues; furthermore, given the subsequent effects on the general status, oncology treatment can trigger difficulties in maintaining oral hygiene [2,3].

In strong relation with periodontal homeostasis, the gingival crevicular fluid (GCF) is regularly a serous transudate and an inflammatory exudate in pathologic conditions, being easily collected from the gingival sulcus. Various components of the GCF can be considered genuine periodontal inflammatory mediators, mostly cytokines, matrix metalloproteinases, or oxidative stress markers: IL-1 (interleukin 1), IL-6 (interleukin 6), IL-17 (interleukin 17), TNF-α (alfa tumoral necrosis factor), TGF-β (beta transforming growth factor), MMP-9 (metalloproteinase 9), SOD (superoxide dismutase), MDA (malondialdehyde), 8-OHdG (8 hydroxy deoxyguanosine), and others.

The literature is abundant with studies focused upon the evaluation of the interconnections between periodontal disease and other systemic conditions like type I and II diabetes, cardiac disorders, or oncologic diseases. In most of the cases reflecting systemic diseases, the biomarkers were analyzed in serum, plasma, or gingival crevicular fluid [4,5,6]. Moreover, the possible connection between the chronic inflammatory status of infectious origin and etiology, like periodontal disease and systemic conditions, remains one of the most challenging issues raised in the dental and scientific community [7].

In the event of immune system alterations, there is also an impairment of the ecological equilibrium in the oral cavity. The inflammation turns chronic over time and leads to alterations of the periodontal tissues, while bacterial shift developed over the course of periodontal disease evolution can be associated with unbalanced inflammatory reactions [8].

The presence of microbial pathogens in periodontal and periapical tissues triggers an initial production of pro-inflammatory cytokines, like IL-1β, that stimulate the expression and activation of matrix metalloproteinases (MMPs), which in turn will degrade the extracellular connective tissue matrix [9]. It seems that bacteria stimulate bone resorption through pro-inflammatory cytokine induction, such as IL-1β, IL-1α, RANKL (receptor activator of nuclear factor kappa beta), or TNF-α [10].

Using antagonists of IL-1 receptors, some authors have proven the role of IL-1 in stimulating periapical bone destruction and thus pointed out significantly higher values of IL-1α in serum and gingival crevicular fluid in patients with periodontal disease compared to healthy ones [11]. Similar results were reported by other researchers, describing higher concentrations of IL-1β in saliva and gingival crevicular fluid of patients with gingivitis and periodontitis compared to healthy ones [12].

In the complex action of the immune response, most studies have associated the presence of IL-1β, along with IL-6, with a pro-inflammatory phase. Elevated values of these cytokines associated with altered periodontal tissues support their determinant role in the evolution of periodontal disease severity [13,14,15].

There are also some controversies in the literature regarding the role of IL-17 in the etiology and pathology of periodontal disease, with some studies not being able to prove an association between the presence of this cytokine and periodontal disease [16,17], while other authors have reported higher serum values of IL-17 in periodontally injured patients compared to a control group [18,19]. Several studies have associated IL-17 with the initiation and progression of periodontal disease through its role in the induction of tissue destruction and bone loss [20,21,22]. Considering the importance of IL-17 expression, most research published prior to the current classification of periodontal diseases adopted in 2017 [23], have associated high levels of this cytokine with what was previously described as chronic periodontitis, even if the severity of clinical manifestations was more important in the periodontal disease previously classified as aggressive periodontitis [24,25].

TGF-β is a multifunctional cytokine, exhibiting pro-inflammatory action through its chemotactic effect on neutrophils, monocytes, mastoidal cells, and lymphocytes, as well as having a role in the release of pro-inflammatory cytokines like IL-1 or IL-6 [26]. Nonetheless, TGF-β also provides an anti-inflammatory role by suppressing the cellular and humoral-mediated immune response [27].

Most studies associate periodontal disease with elevated levels of TGF-β found in gingival tissues, gingival crevicular fluid, and serum [28,29,30,31].

The literature is scarce regarding the correlation between the described cytokines and periodontal disease in children in general, and in children with leukemia in particular, thus raising the particular need for an intimate approach and further research in this direction, in order to achieve proper disease management and intensive reduction of the possible oral consequences of the severe systemic condition.

Our research aimed to correlate the oral health status and certain biological indicators in these patients—namely, the interleukins IL-1β, IL-6, and IL-17α and the regulatory cytokine TGF-β1—which are highly involved in the inflammatory network of gingivo-periodontal tissues in children affected by leukemia. Most of the research trying to reveal the association between inflammatory markers and periodontal disease in the context of a systemic condition are mainly focused on adult patients, and information regarding these interactions in children is relatively scarce [32].

The possibility of an early exploration of the relationship between the level of the mentioned biomarkers and the severity of the gingivo-periodontal alterations in children with leukemia is also pursued in this study. This association would be useful in expressing a certain prognostic role of the biological indicators regarding the complex approach of oral complications management of such severe disease.

## 2. Materials and Methods

Our study was carried out in accordance with the Declaration of Helsinki and was approved by the Ethical Committee of the “Grigore T. Popa” University of Medicine and Pharmacy from Iasi and the Ethical Committee of the “St. Mary” Clinical Emergency Hospital for Children in Iasi.

This study initially included 99 children, aged between 3 and 18 years old, who were enrolled between October 2024 and May 2025 at “St. Mary” Clinical Emergency Hospital for Children in Iasi. The participants were divided into two groups: a study group, *n* = 49 children with confirmed diagnosis of leukemia (28 boys and 21 girls), and a control group, *n* = 50 healthy children (21 boys and 29 girls) without oncological pathology. The inclusion criteria for the study group were children up to 18 years old, with any form of leukemia (acute lymphoblastic, acute myeloid or chronic), in any stage of the antineoplastic treatment (induction, consolidation, maintenance), with or without gingivo-periodontal conditions, who have not undergone any specific periodontal treatment in the previous 3 months. The inclusion criteria for the control group were children up to 18 years of age, without any systemic conditions and who had not encountered any specific periodontal treatment in the previous 6 months. The exclusion criteria for both groups were patients over 18 years of age, or those who had undergone a specific periodontal cure in the previous 6 months. Out of the 99 children enrolled in the study, biological samples were successfully collected for 97 of them, as two children from the study group refused biological sampling. Consequently, the final study group consisted of 47 subjects, while the control group included 50 subjects.

Clinical examination of all subjects was conducted by a periodontal specialist, and the oral hygiene and gingivo-periodontal health status were evaluated using the Oral Hygiene Index (OHI) and Gingival Index (GI). The inflammatory biomarker values (IL-1β, IL-6, IL-17α, and TGF-β1) were analyzed both in plasma and gingival crevicular fluid. GCF samples were collected with calibrated absorbent paper cones, using certain standardized algorithms and steps: 1. Teeth isolation with cotton rolls; 2. Gentle removal of any visible supragingival plaque using cotton rolls; 3. Insertion of the absorbent paper cone in the gingival sulcus, in mesial sites of selected teeth (maxillary or mandibular incisors, maxillary or mandibular first permanent molars) for 30 s; 4. Inserting the paper cones in sterile Eppendorf tubes and transporting them on ice up to the Periotron calibrated device, where the GCF volume was measured; 5. Paper cone insertion in Eppendorf tubes with 200 µL phosphate-buffered saline (PBS) solution and storage at −80 °C until biological markers analysis, subsequent to volume measurement. Peripheral blood samples were obtained through routine blood collection and sampling during the children’s hospitalization, using tubes containing EDTA (ethylenediaminetetraacetic acid). The samples were immediately centrifuged at 1000× *g* at 4 °C for 15 min, and the obtained plasma was aliquoted into Eppendorf tubes and stored at −80 °C until parameter evaluation.

IL-1β, IL-6, IL-17α, and TGF-β1 were measured both in GCF and plasma samples using enzyme-linked immunosorbent assay (ELISA) kits from Elabscience^®^, Bionovation Inc., Houston, TX, USA. Both GCF and plasma samples were thawed at the moment of biomarker analysis, and all the reactive agents and standards were prepared according to the manufacturers’ protocol.

The results were statistically analyzed using the IBM SPSS Statistics 26 software package, which allowed the evaluation of the relationship between variables, comparation of groups, and in-between group variation analysis through ANOVA and post hoc tests.

## 3. Results

### 3.1. Descriptive Data

The age of children included in this study ranged between 3 and 18 years old, with mean values in the control group of 12.16 (±0.51) years, and 8.71(±0.53) years in the study group (Table 1).

**Figure 1:**
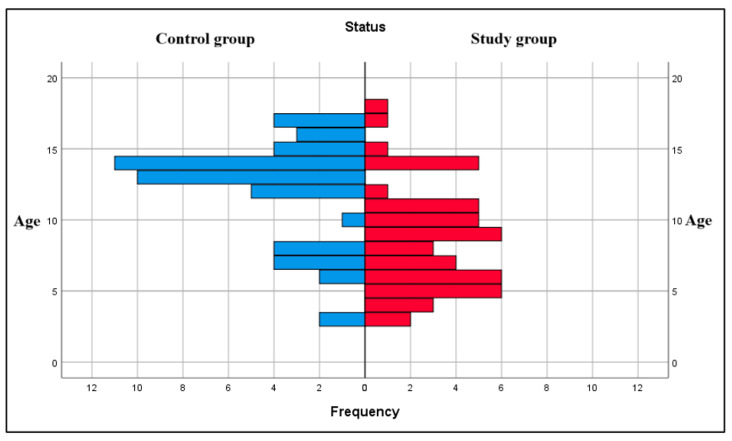
Comparison of age distribution between the groups.

The back-to-back chart below highlights a comparative description of the age distribution of the two groups. The results point out that most children in the control group (healthy) were between 13 and 15 years of age, while most children between 5 and 10 years of age were in the study group (children with leukemia). Thus, it seemed that the study group was formed from a large proportion of young children, while in the control group, adolescents were more prevalent (Figure 1).

The subjects’ distribution according to gender was also analyzed through a contingency table, which pointed out a higher prevalence of male children in the study group compared to females, which were more prevalent in the control group (Table 2).

**Figure 2:**
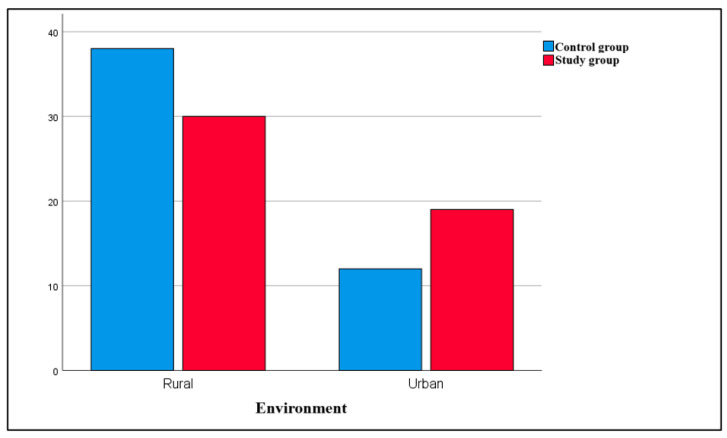
Comparison between groups in terms of their environments.

Regarding the children’s environment, the analysis revealed that most subjects lived in rural areas (68.7% of the total). Moreover, the comparison between groups revealed that most subjects in both the study group (76%) and control group (61.2%) came from rural areas (Figure 2).

The analysis of oral hygiene status by group pointed out that all children with poor oral hygiene status belonged to the study group (seven out of seven cases), children with unsatisfactory oral hygiene were mostly found in the study group (17 out of 26 cases, meaning 65.4%), and children with satisfactory and good oral hygiene status mostly belonged to the control group (58.8% for satisfactory oral hygiene status and 65.6% for good oral hygiene status) (Table 3).

Concerning the evaluation of the gingival inflammatory status by group, our study recorded severe gingival inflammation exclusively in children from the study group (13 out of 13 cases), whereas children with moderate gingival inflammation were mostly within the study group (14 out of 23 cases, meaning 60.9%), and children with mild gingival inflammation belonged mainly to the control group (41 out of 63 cases, therefore 65.1%) (Table 4).

**Figure 3:**
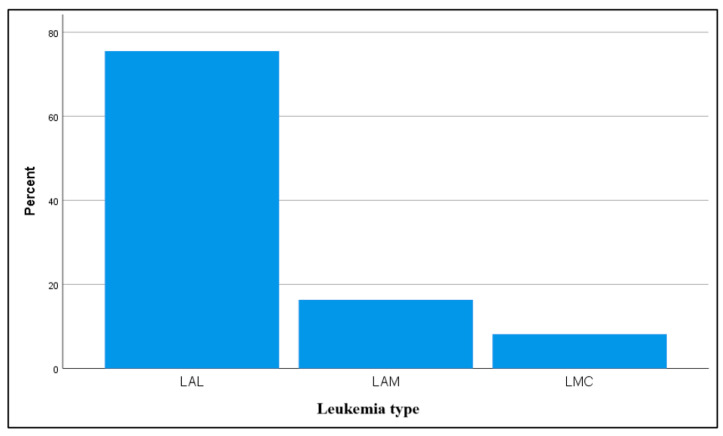
Distribution of children in the study group in terms of leukemia type.

Among the children in the study group, 37 (75.5%) were affected by acute lymphoblastic leukemia (ALL), 8 (16.3%) by acute myeloblastic leukemia (AML), and 4 (8.2%) by chronic myeloid leukemia (CML). This distribution highlights a clear predominance of ALL within the group, as reflected in Figure 3.

Regarding the treatment phase for the enrolled children with leukemia, Table 5 points out the maintenance step as the predominant stage—55.1%, followed by almost a quarter being in the induction phase (24.5%) and 20.4% of subjects during the consolidation step.

### 3.2. Inflammatory Biomarkers Analysis

The mean values of the local (GCF) and systemic (plasma) biological parameters investigated for the study and control subjects are included in Table 6 and Table 7.

The results indicate noticeable differences between subjects in the control and study group across most analyzed markers, with significantly increased values of cytokines in children from the study group, both in GCF and plasma, compared to children in the control group.

Regarding the association between the levels of inflammatory biomarkers and oral hygiene status, most variables in our analysis indicated a decrease in their mean values along with the increase in oral hygiene status, with greater variations observed in the “poor oral hygiene status” group (Table 8). These data suggested a possible association between oral hygiene status and some biomarker values, but without any causal conclusions, thus requiring ANOVA method application.

For most inflammatory biomarkers, the ANOVA analysis results were not statistically significant, but out of the four main biochemical parameters, IL-1β and IL-17⍺ recorded important levels in GCF, suggesting a significant difference between the two main groups. Subsequently, Levene’s test for homogeneity of variances revealed inequal variances between the groups, which is why the Tamhane T2 post hoc test was chosen to further analyze the differences.

The results of the Tamhane T2 tests points out that the differences between mean GCF IL-17⍺ values exceed statistical significance thresholds when analyzed in terms of oral hygiene status groups, while for gingival fluid IL-1β, there were some indicators reflecting a statistically significant difference between the oral hygiene groups in the two extremes (poor versus satisfactory/good status). Our results suggest that oral hygiene status influenced the two biomarkers on different levels (Table 9).

**Figure 4:**
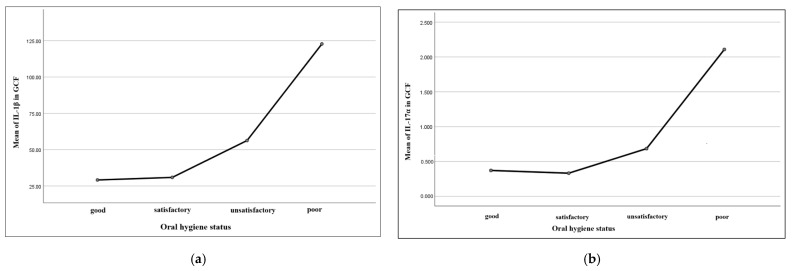
Graphic expressions of the relationship between local (GCF) levels of IL-1β (**a**) and IL-17⍺ (**b**) and oral hygiene status.

The relationship between the analyzed inflammatory biomarkers and oral hygiene status is better expressed through a graphic description, which depicts the increasing values of the cytokines along with the deterioration of the oral hygiene status (Figure 4).

Regarding the association between GCF and plasma inflammatory biomarker values and gingival inflammation status, our results reveal that in most cases, the biomarkers tended to record higher mean values when the gingival inflammation was more prominent (Table 10). This tendency was even more powerful for IL-1β in GCF and plasma, IL-6 in plasma, and IL-17⍺ in GCF. Some high standard deviations in certain groups offered an important base for other statistic tests that were further applied.

The ANOVA test revealed statistically significant differences between groups in the case of IL-1β in GCF (sig = 0.031), IL-6 in plasma (sig = 0.008), and IL-17⍺ in GCF (sig = 0.016), for a significant threshold of 0.05. For plasmatic levels of IL-1β (sig = 0.080) and TGF-β1 (sig = 0.056), statistically significant differences were recorded for a threshold of 0.1. For the other variables, the results were not statistically significant (Table 11).

Subsequent to the ANOVA test, further statistical analysis was performed for the biomarkers where statistically significant differences were achieved—Levene’s test for homogeneity of variances, and consequently, Bonferroni and Tamhane tests. The results showed that only IL-1β and IL-17⍺ in GCF presented statistically significant differences between certain levels of gingival inflammation status, especially between mild and severe inflammation.

**Figure 5:**
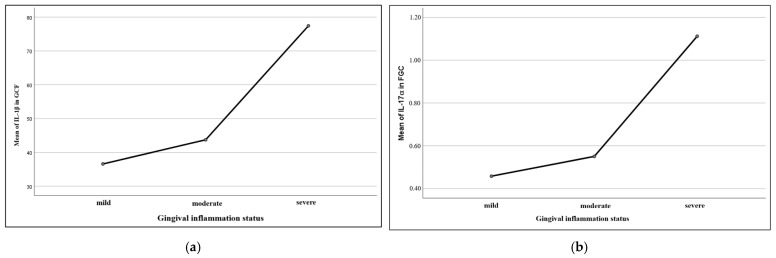
Graphic expression of mean IL-1β values in GCF (**a**) and IL-17⍺ in GCF (**b**) according to gingival inflammatory status.

Our findings are better expressed through the graphics in Figure 5, which illustrate mean values of the biomarkers according to the gingival inflammation status (mild, moderate, severe). In the first graphic (a), we can observe a clear growth of mean gingival fluid IL-1β values along with the gingival inflammation degree: the marker’s mean values gradually increased from mild to moderate inflammation, followed by a sudden increase in the case of severe inflammation, thus suggesting a possible connection between high levels of the interleukin and inflammation severity. In the second graphic (b), it is to be noticed that the mean GCF IL-17⍺ levels remained relatively constant between mild and moderate inflammation, but with a quick augmentation along with severe inflammation. These graphics support the statistical interpretation and offer an intuitive image of the path through which the biological markers can vary according to the severity of the gingival inflammation, consolidating the conclusion that advanced forms of inflammation are associated with higher local values of certain biomarkers.

## 4. Discussion

The literature data describes an association between leukemia and younger age groups [33,34,35] and a gradual increase in acute leukemia in children and adolescents [36,37]. In our research, the mean age of subjects in the study group was 8.71 (±0.527) years, with most children with leukemia being in the 5- to 10-year-old interval, which is similar to other findings [38].

Regarding the gender distribution of subjects in the study group, we found that 57.1% of the children with leukemia were boys and 42.9% were girls, which is somehow very much like other percentages in the literature that claim a relatively higher number of boys being affected by leukemia compared to girls. For example, a study published in 2012 reported that from 15215 children diagnosed with leukemia in the period 1973 to 2006, 56.7% (8622) were boys and 43.3% (6593) were girls [39]. Other authors reported similar results [40,41].

Different studies communicate inconsistent results regarding the relationship between pediatric leukemia and the environment of the child. In our study, most subjects with leukemia came from rural areas (61.22% of children in the study group), consistent with the results of González García et al. in 2018 [42]. At the same time, the environmental distribution has been differently reported by other authors, finding a higher prevalence of leukemia in urban areas, possibly related to the factors associated with urbanization and the proximity to industrial risk factors in metropolitan areas [43,44,45,46].

In terms of leukemia type, our study recorded acute lymphoblastic leukemia (ALL) as the most frequent form, similar to the prevalence addressed by most of the studies [47,48,49].

The literature is limited with respect to oral hygiene status in children with leukemia, but several studies have evaluated this aspect, either through solely descriptive methods or in association with an oral care protocol initiated for these children. Results fluctuate and are usually conflicting, according to the phase of the treatment or if oral care measures were instated or not. For instance, Ponce-Torres et al. found poor oral hygiene in children with acute lymphoblastic leukemia in their study from 2010 [50]. Dholam et al. also reported, in 2014, an alteration of the oral health status in children with leukemia during the induction phase of chemotherapy, with a significant increase in the Oral Hygiene Index values post-chemotherapy (in 50% of the individuals), with further studies being required in order to state whether oral hygiene has a role in the induction outcome of pediatric patients under acute leukemia therapy [51]. A deterioration of oral health status and increased dental caries experience in children with acute lymphoblastic leukemia was observed by Hegde et al. [52], while Kilic et al. [53] also reported a regress in oral health status for children with leukemia.

In our study, in the absence of an established oral care protocol, the oral hygiene status was mostly poor and unsatisfactory in the study group, urging dental preventive management and oral health care measures in children with leukemia, as proposed by other authors. For example, Mazaheri et al. highlighted the necessity of oral hygiene programs for ALL children, given their oral health status [54], similar to other authors who have suggested that oral hygiene care regimens can improve oral health status in children undergoing chemotherapy [55]. Kapoor et al. found that following treatment and an oral care protocol, children with acute lymphoblastic leukemia presented a better oral health status compared to healthy children [56]. Pels and Mielnik-Blaszczak have also described improved oral hygiene status in children with ALL following an oral hygiene regime during oncological treatment protocols but also increased values of gingival inflammation compared to healthy children [57]. Bardellini et al. pointed out a progress in the oral hygiene of children with acute lymphoblastic leukemia following chemotherapy, after the use of a specifically designed toothpaste containing salivary enzymes, essential oils, proteins, and colostrum extract [58]. Sampaio et al. [59] and Galbiati et al. [60] have also reported enhanced oral health outcomes in children with leukemia subsequent to a standardized preventive dental care protocol, emphasizing the need for adaptive, individualized therapeutical management for these children.

Gingival alterations are amongst the most common manifestations of leukemia and its treatment, as documented in two recent meta-analysis [61,62]. Regarding the gingival inflammation observed in subjects from our study, results have shown that children with leukemia usually have a worse gingival state, with mostly severe and moderate gingival inflammation status, as compared to children in the control group. These results are similar to other findings in the literature [50,63,64,65]. Gingival inflammation and periodontal health state could be improved after the implementation of oral health care protocols, as reported by various researchers [56,59].

Similar to our achievements, most authors have found in individuals with malignant hematological diseases an association between the burden of certain inflammation biomarkers and periodontal alterations [61,66]. Results communicated through research and literature are rather scarce when considering the same associations in children with ALL or other cancerous hematological conditions.

IL-1β in saliva and gingival crevicular fluid is considered a relevant marker for periodontal impairments [67,68]. Some authors have proved a pro-inflammatory role of this cytokine in periodontal tissues, suggesting an association between elevated values of oral IL-1β and more severe periodontal conditions [15]. In our study, IL-1β values in gingival crevicular fluid of children with leukemia and gingival alterations were significantly higher with respect to the control group. These results are similar to those included and reported in the limited number of current published studies in children, which claim increased IL-1β values proportionally with periodontal disease severity [69,70].

A recent study evaluated the relationship between certain oral hygiene status parameters, gingival inflammation, and IL-6 in the gingival crevicular fluid in children between 4 and 16 years of age. The authors reported high levels of IL-6, proportionally associated with different degrees of gingival inflammation [71]. The results in our study are similar to these findings, with more elevated values of IL-6, both systemic (in plasma) and GCF, in children with leukemia, compared to the pediatric control group.

Studies evaluating IL-17α values in gingival crevicular fluid are limited, with most authors associating high levels of the cytokine with different severity degrees of periodontal disease in adults [72]. In children, studies usually approached the association between plasma IL-17α levels and certain systemic diseases, such as inflammatory bowel disease, juvenile idiopathic arthritis, or even autism [73,74,75]. Some authors have reported the presence of elevated values of IL-17α in GCF in children under orthodontic treatment, associating cytokine expression with the remodeling bone processes during the orthodontic dental movement [76]. In our study, we observed augmented levels of IL-17α in the gingival crevicular fluid and plasma of children with leukemia, as compared to children in the control group. These results seem to be similar to other literature data available but for the adult population, which proportionally associate these cytokine levels with gingival inflammation of different intensity degrees [77].

TGF-β1 values in children were also mainly studied in association with certain systemic conditions like autism, nephrotic syndrome, or chronic respiratory diseases [78,79,80]. Most studies that observed a relationship between high levels of this cytokine in GCF and periodontal disease were conducted on adult patients [31,81]. Consistent with the mentioned findings, our results describe higher levels of TGF-β1 in gingival crevicular fluid and plasma of children with leukemia, compared to children in the control group.

In summary, most interleukins evaluated in our study seem to present decreasing values in synchrony with a better oral hygiene status and gingival inflammation state, similar to other authors’ findings [71].

The literature is not abundant in research evaluating inflammatory biomarkers in GCF of children with leukemia, but some authors have studied the possibility of salivary cytokines acting like a monitoring factor for pediatric cancer [82]. Furthermore, in their study, Paganini et al. described elevated values of certain cytokines associated with cancer diagnosis, independently from oral health indicators like caries or gingival alterations [82].

Usually, proinflammatory cytokines are studied in association with systemic conditions like obesity, inflammatory bowel disease, diabetes, or others. For example, a study conducted in 2010 reported higher levels of IL-1β in the GCF of obese children, which also presented more gingival inflammation compared to healthy subjects [83]. Other authors also described elevated concentrations of this interleukin in obese children, before and during orthodontic tooth movements, as compared to nonobese adolescents [84]. A recent study reported that IL-17α values could act as a predictor of the increasing severity of periodontal disease in adults with inflammatory bowel disease [85].

The literature seems to be creating a tendency regarding the possibility of using the presence of inflammatory biomarkers in gingival crevicular fluid as prognostic factors for certain diseases. One study that evaluated the association of elevated GCF IL-1β levels with periodontal microinflammation caused by bruxism claimed that the expression of this cytokine could be a potential predictor for the periodontal diagnosis and prevention of further inflammation [86]. A study conducted in 2024 associated high values of IL-6 and IL-17 in GCF with the presence of severe and advanced periodontitis, suggesting a possible diagnostic role of the cytokines in comparing different stages and grades of periodontitis [87]. High values of some systemic inflammatory cytokines (IL-6, IL-8, and TNF-α) in children with leukemia before the initiation of chemotherapy have been reported, and the authors concluded that elevated concentrations of inflammatory biomarkers could be a risk contributor for oral mucositis [88].

Gingival crevicular fluid is thus not only an oral fluid that is found near the gum tissues but one that can be widely used for a proper understanding of the difference between healthy and unhealthy gums. The investigated biomarkers in this fluid can help uncover small changes that develop during gum disease, especially triggered by systemic conditions such as leukemia or other hematologic disorders, making gingival crevicular fluid a suitable tool in distinguishing between healthy and diseased gum conditions.

This study has several limitations that should be acknowledged. First, the narrow sample size might limit the broadening of the rationalization to a higher extent of our findings. Second, no additional statistical adjustment has been performed to isolate the independent impact of different oral hygiene statuses between groups in terms of cytokine levels. Although oral hygiene status could have been influenced by different oral hygiene routines in the two groups, it could also be closely related to the underlying oncological condition of the children in the study group. Various determinants such as fatigue, immunosuppression, or treatment-related soreness may directly affect oral hygiene procedures. We have reported the findings as observed, but adjustment for oral hygiene in this context could avoid bias and guide recognition of genuine association between leukemia and the inflammatory response in the oral environment.

Further perspectives to be followed are related to the development of customized preventive and monitoring protocols tailored specifically for pediatric leukemia patients, in an attempt to abridge gingival and periodontal complications associated with their condition and treatment. Based on our findings, such programs should take into account the unique vulnerabilities of this patient population, including immunosuppression, treatment-related mucosal shift, and reduced oral hygiene capacity. Moreover, further studies are needed to clarify the causal relationships between systemic disease status, oral inflammatory biomarkers, and periodontal health outcomes and prognosis in children with leukemia. These investigations could also evaluate the effectiveness of structured oral care regimens implemented during different phases of the oncological therapy to optimize oral health management in this vulnerable population.

## 5. Conclusions

In our study, we found raised expression of IL-6, IL-17α, and TGF-β1 values both in the plasma and GCF of children with leukemia as compared to healthy subjects, while the levels of IL-1β were elevated only in the GCF. Our findings indicate an association between the levels of these cytokines and both oral hygiene and gingival inflammation status, as higher cytokines levels were correlated with the worsening of oral hygiene status and increasing severity of gingival inflammation. The strongest associations were observed for IL-1β in the GCF and plasma, as well as IL-6 in the plasma, and in IL-17α in the GCF. However, further research is needed to evaluate the relationship between inflammatory biomarkers in the GCF and plasma and oral hygiene status and the gingival inflammation in children with different forms and treatment stages of leukemia, as well as to understand the underlying mechanism driving the interconnection between these conditions.

## Figures and Tables

**Table 1:** Descriptive analysis of age distribution for children in both groups.

Age	Control Group	Study Group
Mean (±SE)	12.16 (±0.51)	8.71 (±0.53)
95% CI for mean	[11.13, 13.19]	[7.66, 9.77]
5% trimmed mean	12.37	8.56
Median	13.00	9.00
Standard deviation (SD)	3.61	3.69
Variance	13.04	13.58
Minimum—maximum	3–17	3–18
Range	14	15
Interquartile range (IQR)	5	5
Skewness (±SE)	–0.93 (±0.34)	0.58 (±0.34)
Kurtosis (±SE)	0.10 (±0.66)	–0.26 (±0.67)

**Table 2:** Distribution of children in terms of gender and group of study.

Gender—Group Crosstabulation					
			Group		Total
			Control	Study	
Gender	Male	Number	21	28	49
		% of Gender	42.9	57.1	100.0
		% of Group	42.0	57.1	49.5
		% of Total	21.2	28.3	49.5
	Female	Number	29	21	50
		% of Gender	58.0	42.0	100.0
		% of Group	58.0	42.9	50.5
		% of Total	29.3	21.2	50.5
Total		Number	50	49	99
		% of Gender	50.5	49.5	100.0
		% of Group	100.0	100.0	100.0
		% of Total	50.5	49.5	100.0

**Table 3:** Distribution of children in terms of oral hygiene status and group.

Oral Hygiene Status and Group Crosstabulation					
			Group		Total
			Control	Study	
Oral hygiene status	poor	Number of cases	0	7	7
		% of Oral hygiene status	0.0	100.0	100.0
		% of Group	0.0	14.3	7.1
		% of Total	0.0	7.1	7.1
	unsatisfactory	Number of cases	9	17	26
		% of Oral hygiene status	34.6	65.4	100.0
		% of Group	18.0	34.7	26.3
		% of Total	9.1	17.2	26.3
	satisfactory	Number of cases	20	14	34
		% of Oral hygiene status	58.8	41.2	100.0
		% of Group	40.0	28.6	34.3
		% of Total	20.2	14.1	34.3
	good	Number of cases	21	11	32
		% of Oral hygiene status	65.6	34.4	100.0
		% of Group	42.0	22.4	32.3
		% of Total	21.2	11.1	32.3
Total		Number of cases	50	49	99
		% of Oral hygiene status	50.5	49.5	100.0
		% of Group	100.0	100.0	100.0
		% of Total	50.5	49.5	100.0

**Table 4:** Distribution of children in terms of gingival inflammation status and group.

Gingival Inflammation Status and Group Crosstabulation					
			Group		Total
			Control	Study	
Gingival inflammation status	mild	Number of cases	41	22	63
		% of Gingival inflammation status	65.1	34.9	100.0
		% of Group	82.0	44.9	63.6
		% of Total	41.4	22.2	63.6
	moderate	Number of cases	9	14	23
		% of Gingival inflammation status	39.1	60.9	100.0
		% of Group	18.0	28.6	23.2
		% of Total	9.1	14.1	23.2
	severe	Number of cases	0	13	13
		% of Gingival inflammation status	0.0	100.0	100.0
		% of Group	0.0	26.5	13.1
		% of Total	0.0	13.1	13.1
Total		Number of cases	50	49	99
		% of Gingival inflammation status	50.5	49.5	100.0
		% of Group	100.0	100.0	100.0
		% of Total	50.5	49.5	100.0

**Table 5:** Distribution of children in the study group in terms of the leukemia type diagnosis (ALL, AML, CML) and treatment stage (induction, consolidation, maintenance).

Leukemia Type	Treatment Phase	Count	% Within Leukemia Type	% Within Treatment Stage	% of Total
ALL	Induction	8	21.6	66.7	16.3
	Consolidation	8	21.6	80.0	16.3
	Maintenance	21	56.8	77.8	42.9
AML	Induction	3	37.5	25.0	6.1
	Consolidation	2	25.0	20.0	4.1
	Maintenance	3	37.5	11.1	6.1
CML	Induction	1	25.0	8.3	2.0
	Consolidation	0	0.0	0.0	0.0
	Maintenance	3	75.0	11.1	6.1
Total		49	100.0	100.0	100.0

**Table 6:** Mean values of inflammatory biomarkers in the GCF and plasma of children in the study group.

	GCF Values				Plasma Values			
Statistical Indicator	IL-1β	IL-6	IL-17⍺	TGF-β1	IL-1β	IL-6	IL-17⍺	TGF-β1
Unit	pg/μL	pg/μL	pg/μL	pg/μL	pg/mL	pg/mL	pg/mL	ng/mL
Mean (SE)	73.15 (8.92)	3.90 (0.41)	0.96 (0.14)	0.65 (0.10)	17.09 (3.33)	11.36 (2.11)	319.61 (43.13)	4.81 (0.53)
95% CI	[55.20, 91.11]	[3.09, 4.72]	[0.68, 1.23]	[0.44, 0.85]	[10.39, 23.80]	[7.11, 15.62]	[232.79, 406.43]	[3.76, 5.87]
Median	55.35	3.20	0.64	0.43	0.00	7.03	232.54	3.90
Variance	3740.10	7.75	0.88	0.50	521.58	210.03	87,441.00	12.95
Standard Deviation	61.16	2.79	0.94	0.71	22.84	14.49	295.70	3.60
Minimum	8.97	0.55	0.24	0.03	0.00	0.54	0.00	0.30
Maximum	254.46	8.79	3.92	3.34	69.02	62.37	825.53	16.70
Range	245.49	8.25	3.67	3.31	69.02	61.83	825.53	16.40
Interquartile range	60.67	5.20	0.53	0.63	30.58	5.52	547.35	3.32
Skewness (SE)	1.38 (0.35)	0.40 (0.35)	2.60 (0.35)	2.38 (0.35)	1.11 (0.35)	2.92 (0.35)	0.42 (0.35)	1.86 (0.35)
Kurtosis (SE)	1.18 (0.68)	−1.37 (0.68)	5.91 (0.68)	6.32 (0.68)	−0.02 (0.68)	8.01 (0.68)	−1.37 (0.68)	4.06 (0.68)

**Table 7:** Comparative description of inflammatory biomarker values in GCF and plasma of children in the study and control groups.

	Group Statistics					
	Inflammatory Biomarker (Unit)	Group	*n*	Mean	Std. Deviation	Std. Error Mean
GCF values	IL-1β (pg/μL)	Control	50	16.09	3.21	0.45
		Study	47	73.15	61.16	8.92
	IL-6 (pg/μL)	Control	50	2.04	0.70	0.10
		Study	47	3.90	2.78	0.41
	IL-17⍺ (pg/μL)	Control	50	0.20	0.08	0.01
		Study	47	0.96	0.94	0.14
	TGF-β1 (pg/μL)	Control	50	0.25	0.20	0.03
		Study	47	0.65	0.71	0.10
Plasma values	IL-1β (pg/mL)	Control	50	17.48	10.71	1.51
		Study	47	17.09	22.84	3.33
	IL-6 (pg/mL)	Control	50	5.31	2.52	0.36
		Study	47	11.36	14.49	2.11
	IL-17⍺ (pg/mL)	Control	50	179.26	210.86	29.82
		Study	47	319.61	295.70	43.13
	TGF-β1 (ng/mL)	Control	50	3.23	1.82	0.26
		Study	47	4.81	3.60	0.52

**Table 8:** Analysis of the associations between the local and systemic inflammatory parameter levels and oral hygiene status.

Fluid	Numeric Variables	Oral Hygiene Status	*n*	Mean	Std. Deviation	Std. Error	95% CI Lower Bound	95% CI Upper Bound	Minimum	Maximum
GCF	IL-1β	poor	7	122.79	72.15	27.27	56.06	189.52	61.33	232.18
		unsatisfactory	26	56.29	59.09	11.59	32.42	80.16	14.31	254.46
		satisfactory	32	30.88	31.07	5.49	19.68	42.08	8.97	147.31
		good	32	29.11	37.24	6.58	15.69	42.54	10.68	179.67
		Total	97	43.74	51.18	5.20	33.43	54.06	8.97	254.46
	IL-6	poor	7	3.38	3.21	1.21	0.41	6.35	0.60	8.23
		unsatisfactory	26	3.59	2.56	0.50	2.56	4.63	0.62	8.64
		satisfactory	32	2.72	1.99	0.35	2.00	3.43	0.77	8.79
		good	32	2.55	1.77	0.31	1.91	3.19	0.55	8.73
		Total	97	2.94	2.20	0.22	2.50	3.39	0.55	8.79
	IL-17⍺	poor	7	2.11	1.65	0.62	0.59	3.63	0.48	3.92
		unsatisfactory	26	0.68	0.74	0.15	0.39	0.98	0.07	3.87
		satisfactory	32	0.33	0.28	0.05	0.23	0.43	0.07	1.19
		good	32	0.37	0.26	0.05	0.28	0.47	0.06	1.21
		Total	97	0.57	0.75	0.08	0.42	0.72	0.06	3.92
	TGF-β1	poor	7	0.56	0.63	0.24	−0.02	1.13	0.11	1.93
		unsatisfactory	26	0.60	0.70	0.14	0.32	0.88	0.04	3.34
		satisfactory	32	0.37	0.32	0.06	0.26	0.49	0.01	1.52
		good	32	0.35	0.56	0.10	0.15	0.56	0.01	3.09
		Total	97	0.44	0.55	0.06	0.33	0.55	0.01	3.34
Plasma	IL-1β	poor	7	21.35	30.51	11.53	−6.87	49.57	0.00	65.29
		unsatisfactory	26	19.70	21.44	4.21	11.04	28.36	0.00	69.02
		satisfactory	32	17.33	14.12	2.50	12.24	22.42	0.00	61.14
		good	32	14.41	13.71	2.42	9.47	19.36	0.00	40.35
		Total	97	17.29	17.56	1.78	13.75	20.83	0.00	69.02
	IL-6	poor	7	13.24	21.40	8.09	−6.55	33.04	0.54	61.10
		unsatisfactory	26	8.15	4.71	0.92	6.25	10.05	2.35	27.15
		satisfactory	32	7.18	10.43	1.85	3.41	10.94	1.87	62.33
		good	32	8.29	11.32	2.00	4.21	12.38	2.56	62.37
		Total	97	8.24	10.64	1.08	6.10	10.39	0.54	62.37
	IL-17⍺	poor	7	267.16	279.52	105.65	8.64	525.67	0.00	786.17
		unsatisfactory	26	307.36	271.46	53.24	197.72	417.01	0.00	781.52
		satisfactory	32	206.65	246.96	43.66	117.61	295.69	0.00	822.99
		good	32	234.69	273.68	48.38	136.02	333.36	0.00	890.61
		Total	97	247.26	263.75	26.78	194.10	300.42	0.00	890.61
	TGF-β1	poor	7	5.77	5.02	1.90	1.12	10.41	2.13	16.70
		unsatisfactory	26	4.66	3.77	0.74	3.13	6.18	0.71	16.70
		satisfactory	32	3.28	1.95	0.35	2.58	3.98	0.31	7.50
		good	32	3.80	2.14	0.38	3.02	4.57	0.30	9.19
		Total	97	4.00	2.92	0.30	3.41	4.59	0.30	16.70

**Table 9:** Results of the Tamhane T2 test for IL-1β in GCF and IL-17⍺ in GCF.

Numeric Variables	Oral Hygiene Status (i)	Oral Hygiene Status (j)	Mean Dif.	Std. Err	Sig.	95% IC L_S_	Upper Bound
IL-1β in GCF	poor	unsatisfactory	66.50	29.63	0.283	−35.04	168.04
		satisfactory	91.91	27.82	0.084	−11.63	195.45
		good	93.68	28.05	0.077	−9.42	196.77
	unsatisfactory	poor	−66.50	29.63	0.283	−168.04	35.04
		satisfactory	25.41	12.83	0.289	−10.29	61.11
		good	27.18	13.33	0.256	−9.69	64.05
	satisfactory	poor	−91.91	27.82	0.084	−195.45	11.63
		unsatisfactory	−25.41	12.83	0.289	−61.11	10.29
		good	1.77	8.57	1.000	−21.56	25.09
	good	poor	−93.68	28.05	0.077	−196.77	9.42
		unsatisfactory	−27.18	13.33	0.256	−64.05	9.69
		satisfactory	−1.77	8.57	1.000	−25.09	21.56
IL-17⍺ in GCF	poor	unsatisfactory	1.42	0.64	0.323	−0.93	3.78
		satisfactory	1.78	0.62	0.162	−0.61	4.16
		good	1.74	0.62	0.175	−0.65	4.12
	unsatisfactory	poor	−1.42	0.64	0.323	−3.78	0.93
		satisfactory	0.35	0.15	0.161	−0.08	0.78
		good	0.31	0.15	0.260	−0.12	0.74
	satisfactory	poor	−1.78	0.62	0.162	−4.16	0.61
		unsatisfactory	−0.35	0.15	0.161	−0.78	0.08
		good	−0.04	0.07	0.993	−0.22	0.15
	good	poor	−1.74	0.62	0.175	−4.12	0.65
		unsatisfactory	−0.31	0.15	0.260	−0.74	0.12
		satisfactory	0.04	0.07	0.993	−0.15	0.22

**Table 10:** Associations between inflammatory biomarkers and gingival inflammation status.

Fluid	Numeric Variables	Gingival Inflammation Status	*n*	Mean	Std. Deviation	Std. Error	95% CI Lower Bound	95% CI Upper Bound	Minimum	Maximum
GCF	IL-1β	mild	61	36.57	50.95	6.52	23.52	49.62	8.97	254.46
		moderate	23	43.72	45.68	9.53	23.97	63.47	13.16	179.67
		severe	13	77.42	51.57	14.30	46.26	108.58	10.68	179.11
		total	97	43.74	51.18	5.20	33.43	54.06	8.97	254.46
	IL-6	mild	61	2.71	1.93	0.25	2.22	3.21	0.55	8.79
		moderate	23	3.51	2.52	0.53	2.42	4.60	1.12	8.64
		severe	13	3.04	2.77	0.77	1.36	4.71	0.60	8.73
		total	97	2.94	2.20	0.22	2.50	3.39	0.55	8.79
	IL-17⍺	mild	61	0.46	0.69	0.09	0.28	0.63	0.06	3.92
		moderate	23	0.55	0.74	0.15	0.23	0.87	0.07	3.78
		severe	13	1.11	0.89	0.25	0.57	1.65	0.25	3.87
		total	97	0.57	0.75	0.08	0.42	0.72	0.06	3.92
	TGF-β1	mild	61	0.38	0.46	0.06	0.26	0.50	0.01	3.09
		moderate	23	0.49	0.53	0.11	0.26	0.72	0.01	1.94
		severe	13	0.64	0.87	0.24	0.11	1.17	0.04	3.34
		total	97	0.44	0.55	0.06	0.33	0.55	0.01	3.34
Plasma	IL-1β	mild	61	15.09	12.75	1.63	11.82	18.35	0.00	40.35
		moderate	23	17.60	18.93	3.95	9.41	25.78	0.00	69.02
		severe	13	27.09	29.49	8.18	9.27	44.91	0.00	64.81
		total	97	17.29	17.56	1.78	13.75	20.83	0.00	69.02
	IL-6	mild	61	6.19	4.41	0.57	5.06	7.32	1.87	32.45
		moderate	23	9.33	12.37	2.58	3.98	14.68	2.17	61.10
		severe	13	15.95	20.96	5.81	3.28	28.61	0.54	62.37
		total	97	8.24	10.64	1.08	6.10	10.39	0.54	62.37
	IL-17⍺	mild	61	229.85	244.11	31.26	167.33	292.37	0.00	890.61
		moderate	23	275.27	284.27	59.27	152.35	398.20	0.00	825.53
		severe	13	279.41	325.97	90.41	82.42	476.39	0.00	786.17
		total	97	247.26	263.75	26.78	194.10	300.42	0.00	890.61
	TGF-β1	mild	61	3.67	1.86	0.24	3.20	4.15	0.31	9.19
		moderate	23	3.85	2.89	0.60	2.60	5.10	0.59	13.30
		severe	13	5.79	5.62	1.56	2.39	9.19	0.30	16.70
		total	97	4.00	2.92	0.30	3.41	4.59	0.30	16.70

**Table 11:** ANOVA test applied for inflammatory biomarkers and gingival inflammation status.

	ANOVA						
Fluid	Marker	Groups	Sum of Squares	Df	Mean Square	F	Sig.
GCF	IL-1β	Between groups	17,878.31	2	8939.16	3.60	0.031
		In groups	233,540.57	94	2484.47		
		Total	251,418.88	96			
	IL-6	Between groups	10.66	2	5.33	1.10	0.336
		In groups	453.77	94	4.83		
		Total	464.43	96			
	IL-17⍺	Between groups	4.59	2	2.30	4.32	0.016
		In groups	49.94	94	0.53		
		Total	54.53	96			
	TGF-β1	Between groups	0.80	2	0.40	1.34	0.267
		In groups	28.01	94	0.30		
		Total	28.81	96			
Plasma	IL-1β	Between groups	1547.21	2	773.61	2.59	0.080
		In groups	28,069.77	94	298.61		
		Total	29,616.98	96			
	IL-6	Between groups	1056.08	2	528.04	5.06	0.008
		In groups	9804.25	94	104.30		
		Total	10,860.32	96			
	IL-17⍺	Between groups	49,969.70	2	24,984.85	0.35	0.703
		In groups	6,628,274.46	94	70,513.56		
		Total	6,678,244.17	96			
	TGF-β1	Between groups	48.73	2	24.36	2.98	0.056
		In groups	769.75	94	8.19		
		Total	818.47	96			

## Data Availability

The original contributions presented in this study are included in the article. Further inquiries can be directed to the corresponding author.

## Abbreviations

The following abbreviations are used in this manuscript:

IL	Interleukins
TGF	Transforming growth factor
GCF	Gingival crevicular fluid
ELISA	Enzyme-linked immunosorbent assay
TNF	Tumoral necrosis factor
MMP	Metalloproteinase
SOD	Superoxide dismutase
MDA	Malondialdehyde
8-OHdG	8-Hydroxy deoxyguanosine
RANKL	Receptor activator of nuclear factor kappa beta
OHI	Oral hygiene index
GI	Gingival index
PBS	Phosphate saline buffer solution
EDTA	Ethylenediaminetetraacetic acid
